# Sex-Specific Differences in Mortality of Patients with a History of Bariatric Surgery: a Nation-Wide Population-Based Study

**DOI:** 10.1007/s11695-021-05763-6

**Published:** 2021-11-09

**Authors:** Hannes Beiglböck, Eric Mörth, Berthold Reichardt, Tanja Stamm, Bianca Itariu, Jürgen Harreiter, Miriam Hufgard-Leitner, Paul Fellinger, Jakob Eichelter, Gerhard Prager, Alexander Kautzky, Alexandra Kautzky-Willer, Peter Wolf, Michael Krebs

**Affiliations:** 1 Division of Endocrinology and Metabolism, Department of Internal Medicine III, Medical University of Vienna, Währinger Gürtel 18-20, 1090 Vienna, Austria; 2 Department of Informatics, University of Bergen, 5008 Bergen, Norway; 3 Mohn Medical Imaging and Visualization Centre, Haukeland University Hospital, 5021 Bergen, Norway; 4 Austrian Social Health Insurance Fund, 7000 Eisenstadt, Austria; 5 Institute for Outcomes Research, Center for Medical Statistics, Informatics and Intelligent Systems, Medical University of Vienna, Währinger Gürtel 18-20, Vienna, 1090 Austria; 6 Department of General Surgery, Division of Visceral Surgery, Medical University of Vienna, Währinger Gürtel 18-20, 1090 Vienna, Austria; 7 Department of Psychiatry and Psychotherapy, Division of Social Psychiatry, Medical University of Vienna, Währinger Gürtel 18-20, 1090 Vienna, Austria

**Keywords:** Bariatric surgery, Sex differences, Mortality, Population-based registry analysis, Comorbidities, Healthcare research

## Abstract

**Purpose:**

Bariatric surgery reduces mortality in patients with severe obesity and is predominantly performed in women. Therefore, an analysis of sex-specific differences after bariatric surgery in a population-based dataset from Austria was performed. The focus was on deceased patients after bariatric surgery.

**Materials and Methods:**

The Austrian health insurance funds cover about 98% of the Austrian population. Medical health claims data of all Austrians who underwent bariatric surgery from 01/2010 to 12/2018 were analyzed. In total, 19,901 patients with 107,806 observed years postoperative were eligible for this analysis. Comorbidities based on International Classification of Diseases (ICD)-codes and drug intake documented by Anatomical Therapeutical Chemical (ATC)-codes were analyzed in patients deceased and grouped according to clinically relevant obesity-associated comorbidities: diabetes mellitus (DM), cardiovascular disease (CV), psychiatric disorder (PSY), and malignancy (M).

**Results:**

In total, 367 deaths were observed (1.8%) within the observation period from 01/2010 to 04/2020. The overall mortality rate was 0.34% per year of observation and significantly higher in men compared to women (0.64 vs. 0.24%; *p* < 0.001(Chi-squared)). Moreover, the 30-day mortality was 0.19% and sixfold higher in men compared to women (0.48 vs. 0.08%; *p* < 0.001). CV (82%) and PSY (55%) were the most common comorbidities in deceased patients with no sex-specific differences. Diabetes (38%) was more common in men (43 vs. 33%; *p* = 0.034), whereas malignant diseases (36%) were more frequent in women (30 vs. 41%; *p* = 0.025).

**Conclusion:**

After bariatric surgery, short-term mortality as well as long-term mortality was higher in men compared to women. In deceased patients, diabetes was more common in men, whereas malignant diseases were more common in women.

**Graphical abstract:**

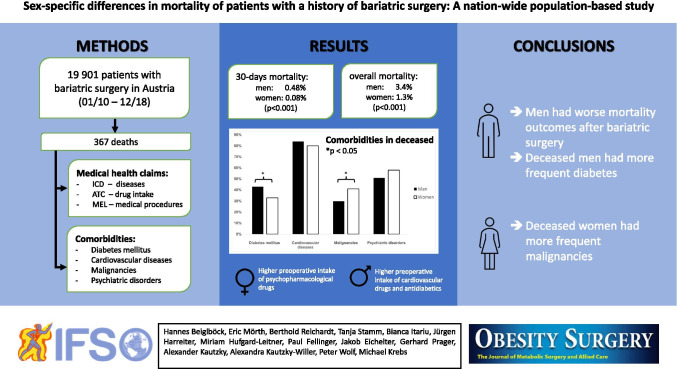

## Introduction

Bariatric surgery is a frequently performed procedure in patients with severe obesity with worldwide increasing numbers [1, 2]. According to the International Federation for the Surgery of Obesity and Metabolic Disorders (IFSO) survey 2016, 634,897 primary bariatric surgeries were performed worldwide, of which 2983 bariatric procedures were done in Austria [3].

Bariatric surgery was shown to lead to substantial weight-loss and to a reduction in all-cause mortality compared to controls with severe obesity [4, 5]. However, a recent study indicates that the mortality after bariatric surgery remains higher compared to the general population [6]. According to the IFSO report 2018, the proportion of female bariatric surgery candidates was 73.7% and remarkably higher compared to men [7]. There are several reasons and motivations for this inequality including psychosocial factors and a stronger desire for weight loss in women [8]. The appearance was shown to be the major motivation for women to undergo bariatric surgery, whereas in men, the medical condition is the most important driving factor [9]. Thus, men undergoing bariatric surgery tend to be older and to have more comorbidities at the time of operation compared to women [10]. Moreover, it was previously shown that male patients might have a higher early mortality rate after bariatric surgery compared to female patients [10, 11]. A study including about 10,000 patients after bariatric surgery showed sex differences regarding preoperative comorbidities and postoperative complications [10].

Potential sex-specific differences in morbidity and mortality are of major importance in daily clinical routine. However, large registry analyses with focus on sex-specific differences after bariatric surgery are rare and selection bias might be a relevant shortcoming in previous studies. Here, an analysis of about 20,000 patients with a history of bariatric surgery and a mean follow-up of more than 5 years is presented. Since medical health claims data of all Austrians are included, a relevant selection bias can be excluded. The aim was to study sex-specific differences in a large, real-world registry analysis.

## Research Design and Methods

The health insurance system in Austria provides healthcare services for all residents who are assigned to one of the several health insurance funds according to their employment or province of residence. Thus, approximately 98% of all Austrians (2018; 8.9 million [12]) are covered. Data from outpatient and inpatient medical services covered by the health insurance funds are stored in the databases of the Austrian health insurance funds. These data include demographic data, reimbursed drug prescriptions based on Anatomical Therapeutic Chemical (ATC)-codes, and along with discharge dates from hospitals, primary, and secondary medical diagnosis recorded as International Classification of Disease (ICD)-codes and medical procedures recorded as MEL-codes (medical single procedure). Moreover, mortality data are recorded in these databases. Furthermore, as the database comprises accounting data of the Austrian health insurance funds, only publicly funded surgeries are included. The study protocol was approved by the ethical committee of the Medical University of Vienna (EK-Nr. 2052/2018).

### Included Patients

All patients with a history of bariatric surgery performed between January 1st, 2010, and December 31st, 2018, based on the following MEL-codes were included in this study: HF220 (sleeve gastrectomy—open), HF230 (sleeve gastrectomy – laparoscopic), HF240 (gastric bypass – open), HF250 (gastric bypass – laparoscopic), HF254 (biliopancreatic diversion – open), HF255 (biliopancreatic diversion – laparoscopic), HF260 (gastric banding – open), and HF270 (gastric banding – laparoscopic).

### Comorbidity Categories

Comorbidities of patients who died during the observation period were analyzed. Based on MEL-codes, ICD-codes, and drug histories (ATC-codes), patients were manually grouped according to the most common and clinically relevant obesity-associated comorbidities: diabetes mellitus, cardiovascular diseases, malignancy, and psychiatric disorders. The psychiatric disorder group comprised patients with schizophrenia, depression, alcohol abuse, drug abuse, anxiety disorders, borderline disorders, and eating disorders and/or took psychopharmacological medications used for treatment of these diseases. The diabetes group comprised patients diagnosed with all types of diabetes and/or took blood glucose-lowering medication. The malignancy group included patients diagnosed with malignant disease and/or took associated medication. Patients diagnosed with heart disease, heart failure, cardiac arrythmia, pulmonary disease, arterial/pulmonal hypertension, peripheral artery disease, and thromboembolic event and/or took related drugs were assigned to the cardiovascular disease group. Moreover, the number of days hospitalized and the number of hospitalizations pre- and postoperatively was analyzed in all deceased patients within the observation period. Normalization of the data was performed by dividing the number of days hospitalized and the number of hospitalizations by the individual observation period in years. For the analysis of the ATC-codes, the following substance classes were included: N03A – antiepileptics, N05A – antipsychotics, N05B – anxiolytics, N06A – antidepressants, N07B – drugs used in addictive disorders, M05B – drugs affecting bone structure and mineralization, M01A – anti-inflammatory and antirheumatic products (non-steroids), A10A – insulin and analogues, A10B – blood glucose-lowering drugs (excluding insulins), C02 – antihypertensives, C03 – diuretics, C04 – peripheral vasodilators, C07 – beta blocking agents, C08 – calcium channel blockers, C09 – agents acting on the renin-angiotensin system, C10 – lipid modifying agents, C10AA – statins, and C10AB – fibrates.

### Sex-Specific Analysis

Mortality rates, comorbidities, number of days hospitalized, number of hospitalizations, drug intake, frequencies of different bariatric procedures, causes of deaths, and rate of revision operations in men and women were studied. Revision operations were identified by a further MEL-code of bariatric surgery recorded after the initial operation. Furthermore, an analysis of preoperative medication in men and women was performed. The overall mortality rate was calculated as the number of deaths divided by the total years of observation. Sex-specific mortality rate was calculated by division of the sex-specific numbers of death by the sex-specific years of observation.

### Statistical Analysis

R (https://www.r-project.org/) was used to combine the datasets from the different institutions and to summarize the data of patients who were insured by multiple Austrian health insurances. Moreover, the ICD-, ATC-, and MEL-codes were linked with their respective lookup tables to enable manual classification of the patient’s comorbidities. Exploratory statistical analysis was performed using SPSS (IBM, version 26) and Microsoft Excel (Microsoft, 2019). Distribution of data was checked by visualization using histograms. Normal distributed data are given in mean ± standard deviation and data not normal distributed are given in median and interquartile range (IQR). Unpaired *t*-tests were performed for normal distributed data whereas for data not normal distributed, Mann–Whitney *U*-tests were used. Chi-squared tests were calculated to compare data between the different subgroups. Moreover, a logistic regression model in all patients (*n* = 19.901) was fitted. Survival/death were used as outcome and gender, age at index surgery, type of surgery, and medication category before the index surgery were used as explanatory variables. Due to the exploratory nature of the study, corrections for multiple testing were not implemented. Data is given as means ± standard deviation. Statistical significance level was set at *p* < 0.05.

## Results

In total, 19,901 patients (5220 men (26%), 14,681 women (74%)) with 107,806 postoperative years of observation were eligible for this analysis. The individual observation period of each enrolled patient ranged from 1 year prior to the date of the bariatric surgery until 04/2020.

### Total Group and Differences Between Men and Women (Table 1)

**Table 1:**
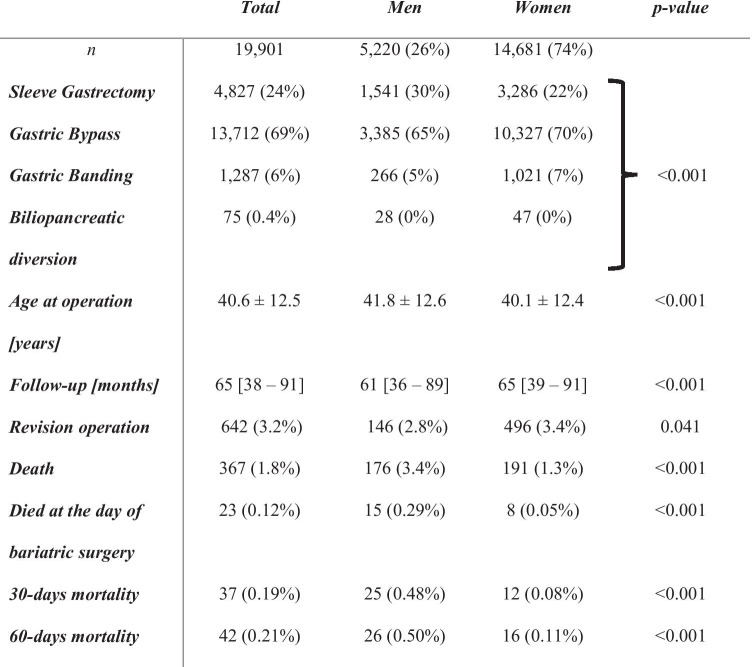
Demographics of Austrian patients with a history of bariatric surgery from 01/2010 to 12/2018

*p*-value represents men vs. women; Data are given in mean ± standard deviation for normal distributed data and in median and interquartile range (IQR) for data not normal distributed

Median follow-up of the total cohort was 65 (IQR 38–91) months (Table 1). The age at operation was significantly higher in men compared to women (41.8 ± 12.6 vs. 40.1 ± 12.4; *p* < 0.001). Gastric bypass (69%) was the most frequently performed procedure in men and women but with a higher proportion in women (65 vs. 70%; *p* < 0.001). Overall, sleeve gastrectomy was recorded in 24% and significantly more often performed in men compared to women (30 vs. 22%; *p* < 0.001). The rate of revision operations was significantly higher in women compared to men (3.4 vs. 2.8%; *p* = 0.04). In patients with revision surgery, sleeve gastrectomy (40%) was the most frequently performed initial type of procedure followed by gastric banding (34%) and gastric bypass (25%). After the revision surgery, gastric bypass was the most frequently performed type of surgery with 79% followed by sleeve gastrectomy (11%) and gastric banding (10%).

### Analysis of Drug Intake Based on ATC-Codes in the Total Group (Table 2)

**Table 2:** Intake of different drugs based on Anatomical Therapeutical Chemical (ATC)-codes in the year before the bariatric surgery in all included patients

*n*	***Total***	***Men***	***Women***	***p-value***
	19,901	5220	14,681	
***Antidepressants***	2367 (12%)	457 (9%)	1910 (13%)	< 0.001
***Antipsychotics***	641 (3%)	149 (3%)	492 (3%)	0.081
***Anxiolytics***	362 (1%)	71 (1%)	291 (2%)	0.004
***Antiepileptics***	595 (3%)	150 (3%)	445 (3%)	0.566
***Addictive disorders***	51 (0.3%)	23 (0.4%)	28 (0.2%)	0.002
***Bone mineralization***	49 (0.2%)	12 (0.2%)	37 (0.3%)	0.782
***Non-steroidal antirheumatics***	4319 (22%)	1092 (21%)	3227 (22%)	0.110
***Insulin***	339 (2%)	147 (3%)	192 (1%)	< 0.001
***Antidiabetics*** ***(without insulin)***	1238 (6%)	479 (9%)	759 (5%)	< 0.001
***Antihypertensives***	376 (2%)	156 (3%)	220 (2%)	< 0.001
***Diuretics***	591 (3%)	215 (4%)	376 (3%)	< 0.001
***Peripheral vasodilators***	44 (0.2%)	18 (0.3%)	26 (0.2%)	0.027
***Beta blocking agents***	1220 (6%)	435 (8%)	785 (5%)	< 0.001
***Calcium channel blocker***	444 (2%)	182 (4%)	262 (2%)	< 0.001
***Renin-angiotensin inhibitors***	2771 (14%)	1099 (21%)	1672 (11%)	< 0.001
***Lipid-lowering***	1376 (7%)	572 (11%)	804 (6%)	< 0.001
***Statins***	1,247 (6%)	516 (10%)	731 (5%)	< 0.001
***Fibrates***	127 (0.6%)	58 (1.1%)	69 (0.5%)	< 0.001

*p*-value represents men vs. women

Medication intake during the year before the bariatric surgery was analyzed. Men were treated significantly more frequently with glucose-lowering drugs including insulin, antihypertensive agents, and lipid-lowering drugs, whereas the intake of antidepressants was significantly higher in women (Table 2).

### *Characterization of Deceased Patients (*Table 3*)*

**Table 3:**
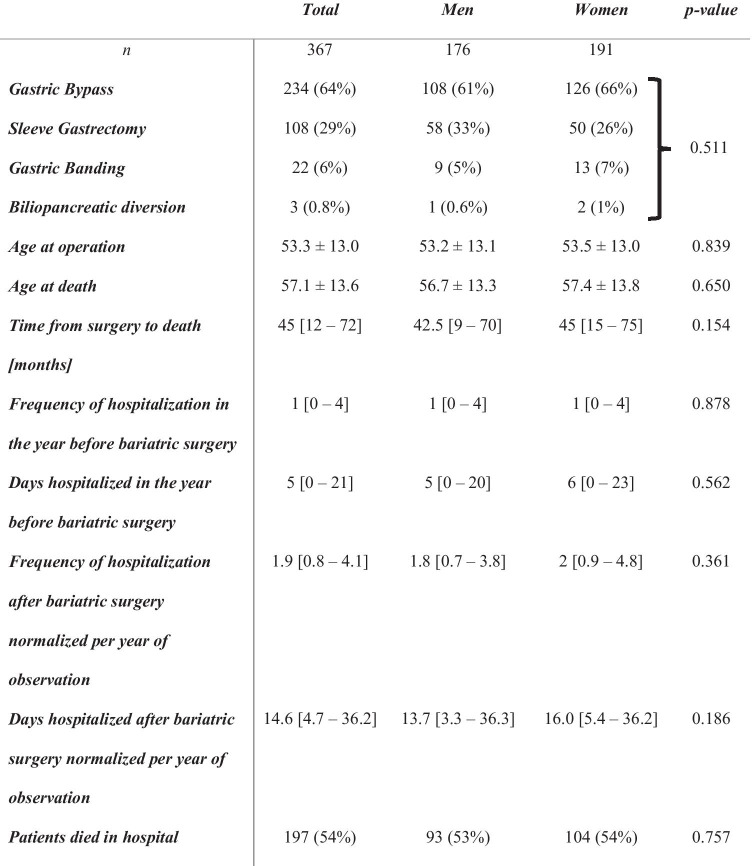
Demographics of deceased patients with a history of bariatric surgery

*p*-value represents men vs. women; Data are given in mean ± standard deviation for normal distributed data and in median and interquartile range (IQR) for data not normal distributed

A total of 367 (1.8%) patients died during the observation period with a significantly higher proportion of men compared to women (3.4 vs. 1.3%; *p* < 0.001) (Table 1). The overall mortality was 0.34% per year of observation and was significantly higher in men compared to women (0.64 vs. 0.24%, *p* < 0.001 (Chi-squared)). Moreover, the proportion of patients who deceased on the day of the bariatric procedure was 0.12% and significantly higher in men compared to women (0.29 vs. 0.05%, *p* < 0.001) (Table 1). The postoperative 30-day mortality was 0.19% and sixfold higher in men compared to women (0.48 vs. 0.08%, *p* < 0.001). Overall, 330 deaths were observed after 30 days postoperative, with a higher proportion of men compared to women (2.9 vs. 1.2%, *p* < 0.001). The overall mortality rate per year of observation excluding the 30-day mortality was 0.31% with a more than twofold higher mortality rate in men (0.55 vs. 0.22%). Five patients (1 man, 4 women, *p* = 0.754) died between day 31 and day 60. Gastric bypass surgery was the most frequently performed procedure in all patients deceased (64%) followed by sleeve gastrectomy (29%) and gastric banding (6%), with no sex-specific differences.

Mean age at operation in deceased patients and mean age at death were not significantly different between men and women. Detailed demographics of deceased subjects are given in Table 3. In total, 197 of 367 patients (54%) died in hospital with no significant sex-specific difference. For these patients, potential causes of deaths, based on the ICD-codes recorded on or around the date of the death, are given in Table 4. No sex-specific differences were observed (*p* = 0.409).

**Table 4:** Causes of deaths (based on ICD-diagnoses) of patients with a history of bariatric surgery and in-hospital death (*p*-value men vs. women = 0.409)

*n*	***Total***	***Men***	***Women***
	197	93	104
***Accident***	1 (0%)	1 (1%)	0 (0%)
***Heart disease***	28 (14%)	13 (14%)	15 (14%)
***Neurological disease***	10 (5%)	4 (4%)	6 (6%)
***Cancer related death***	51 (26%)	26 (28%)	25 (24%)
***Liver failure***	15 (8%)	5 (5%)	10 (10%)
***Kidney failure***	2 (1%)	2 (2%)	0 (0%)
***Shock***	12 (6%)	9 (10%)	3 (4%)
***Infection***	44 (22%)	16 (17%)	28 (27%)
***Embolism***	7 (4%)	4 (4%)	3 (3%)
***Respiratory failure***	19 (10%)	10 (11%)	9 (9%)
***Unknown***	8 (4%)	3 (3%)	5 (5%)

The analysis of the frequency of hospitalizations as well as the number of days hospitalized showed no sex-specific differences neither preoperatively nor postoperatively.

**Fig. 1:**
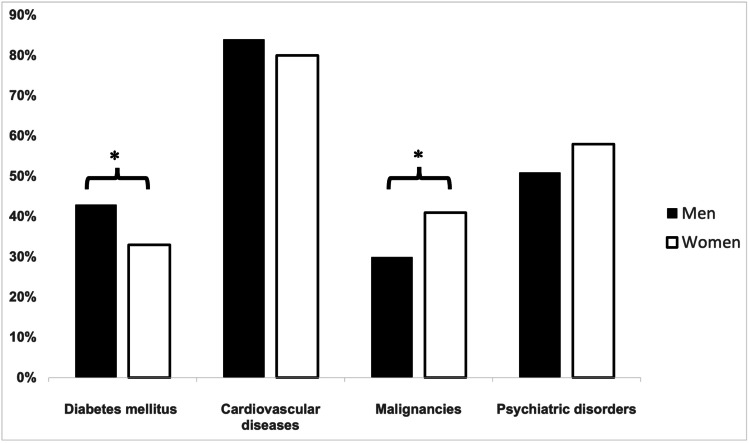
Comorbidities in deceased patients after bariatric surgery. * represents *p* < 0.05 men vs. women; black bars = men; white bars = women

Underlying comorbidities of all 367 patients deceased are given in Fig. 1. Cardiovascular diseases (total 82%, men 84%, women 80%; *p* = 0.332) and psychiatric disorders (total 55%, men 51%, women 58%; *p* = 0.147) were the most common comorbidities with no significant sex-specific differences. Malignant diseases (total 36%, men 30%, women 41%; *p* = 0.025) were significantly more observed in women, whereas diabetes (total 38%, men 43%, women 33%; *p* = 0.034) was more frequent in men. A total of 93% of all patients deceased had at least one comorbidity with no sex-specific difference regarding the number of comorbidities (*p* = 0.909). In 33 deceased patients (9%; men 17, women 16; men vs. women; *p* = 0.668), no definitive comorbidity could be identified. However, all available data on demographics including sex, age, type of bariatric procedure, and date of death were analyzed in these patients.

The logistic regression analysis demonstrated that men (odds ratio 2.4), patients with a higher age at the index surgery (odds ratio 1.1), and patients with preoperative intake of antihypertensive agents (odds ratio 1.6) had a significantly higher risk for death. Preoperative intake of lipid lowering drugs (odds ratio 0.6) and preoperative intake of non-steroidal antirheumatics (odds ratio 0.5) was associated with a significantly lower risk for death.

## Discussion

This analysis of medical insurance claims data shows that (i) short-term mortality as well as long-term mortality after bariatric surgery is significantly higher in men compared to women and (ii) that in patients deceased after bariatric surgery, cardiovascular and psychiatric comorbidities were the most common comorbidities with no sex-specific differences. Moreover, in deceased patients, the incidence of diabetes was higher in men, whereas women suffered more frequently from malignancies. Our results highlight the importance of an individualized, sex-specific preoperative counseling and postoperative follow-up of patients after bariatric surgery.

Several studies demonstrated the favorable outcomes after bariatric surgery including a reduction in overall-mortality [4, 5, 13] reduced hospitalization rates and reduced comorbidities [14]. On the contrary, other observations showed an increased risk of drug- and alcohol-associated mortality [15] and a higher suicide risk after metabolic surgery [16]. However, sex-specific outcomes are not comprehensively analyzed but might be of major importance as the barrier to undergo bariatric surgery is higher in men compared to women, which is reflected by the predominance of females among bariatric surgery candidates [7].

The 30-day mortality and 60-day mortality in our analysis was 0.19% and 0.21%, respectively. This is lower compared to others that reported a 30-day mortality rate of up to 1.1%, depending on the procedure performed [17, 18] and a 60-day mortality rate following bariatric surgery of 0.25% [19]. However, recent studies described a lower 30-day mortality with 0.1% [20]. The 30-day mortality in our analysis over the observation period was stable (range 0.1–0.3%) with almost constantly higher mortality in men compared to women. Sex-specific mortality in men (0.64% per year of observation) was more than two times higher compared to women (0.24%). These results are consistent with the findings from an analysis showing that male sex, higher age, higher body mass index, and psychiatric disorders are linked to higher mortality following bariatric surgery [21].

It was shown that men are more often suffering from comorbidities and have a higher body mass index at the time of the operation compared to women [10]. Also, in our study, the intake of cardiovascular and glucose-lowering medication in the year before the operation was higher in men (Table 2). The higher incidence of diabetes in men observed in this analysis is in line with the literature [10] and of major interest, since it was shown that preoperative diabetes mellitus and hypertension are associated with higher mortality risks in patients with bariatric surgery [19]. However, the overall preoperative intake of antidiabetics and insulin, based on ATC-codes, was 8% and might indicate a low rate of diabetics in this cohort. Nevertheless, this low rate might be an underestimation of the diabetes rate, since only reimbursed drug prescriptions were available. Moreover, the higher age at the operation in men in this analysis is in line with findings from other studies [10, 22] and might also explain the higher postoperative mortality rates in males.

One other potential explanation for the overall better outcomes in women after bariatric surgery might be that the adaptation on protein catabolism occurring during the third trimester of pregnancy [23] might prepare women better for the state of catabolism after bariatric surgery. However, the rate of revision operation in our analysis was significantly higher in women compared to men (3.4 vs. 2.8%, *p* = 0.04). This finding is in line with the literature showing that revision surgery rates in women are remarkably higher compared to men [8, 24]. Nevertheless, the overall revision rate observed in our analysis was lower compared to other studies [25].

The detailed analysis of all deceased patients with a history of bariatric surgery in this study revealed that cardiovascular comorbidities and psychiatric disorders were the most common findings with no sex-specific differences. This is of special interest since it was shown that depression disorder and coronary heart disease are linked to higher postoperative mortality [21, 26]. Moreover, the increased risk of major depression disorders following bariatric surgery [27] might provide one explanation for the high percentage of psychiatric comorbidities observed in our analysis. Regarding sex-specific disease risk, it was shown that women are more likely to have mental health issues before bariatric surgery, whereas men have higher rates of substance abuse and cardiopulmonary diseases [22]. These findings are in line with the results of our analysis, showing that women more frequently use psychopharmacological drugs before bariatric surgery while usage of cardiovascular drugs, glucose-lowering drugs, and drugs used for treatment of addiction disorders was observed predominantly in men. Moreover, it has been shown that men exerted an unhealthier lifestyle after bariatric surgery including higher alcohol intake and a worse macronutrient intake while exceeding recommended calorie intake [28]. However, in our analysis, malignant diseases were significantly more prevalent in women compared to men. Nevertheless, a meta-analysis demonstrated a favorable effect of bariatric surgery on overall obesity-related cancer rates with greater reduction in women compared to men [29].

The pattern of different bariatric procedures observed in this study cohort is different from the worldwide reported frequencies of metabolic procedures [7], since traditionally gastric bypass is performed more frequently than sleeve gastrectomy in Austria. In addition, a sex-specific difference between the frequency of sleeve gastrectomy and gastric bypass was observed in this analysis. Sleeve gastrectomy was more frequent in men, whereas gastric bypass was performed more often in women. This is of interest, since it was shown that sleeve gastrectomy is more effective in men and gastric bypass is neutral concerning sex-specific outcomes [30]. A meta-analysis demonstrated a higher surgery-associated mortality in observational studies after gastric bypass compared to sleeve gastrectomy [25]. However, this might not explain the different mortality rates in men and women observed in this study, since gastric bypass was more commonly performed in women in the present analysis. Nevertheless, in deceased patients, no sex-specific differences regarding bariatric procedures could be observed in our analysis.

The major limitation of this study is the structure of patient’s data based on medical insurance claims data which only comprises ICD-codes, ATC-codes, and MEL-codes. Detailed information on body mass index, weight reduction or gain, metabolic parameters, and diagnoses from family doctors exceeding drug prescriptions are lacking due to the medical insurance health claims data structure. Moreover, potential coding discrepancies between the medical insurance claims data and the real disease must be considered. Furthermore, no data on suicide was available which might be a relevant information since previous studies reported increased suicide rates after bariatric surgery especially for men, underlying psychiatric disorders, and after gastric bypass surgery [16, 31].

On the other hand, the strength of this analysis is the sample size of nearly 20,000 patients with a history of bariatric surgery. Moreover, medical insurance claims data provide a real-world setting including ICD-diagnoses and MEL-codes from hospitals across the whole country as well as information on drug prescription by ATC-codes outside the hospital. As the medical health insurances in Austria are covering more than 98% of all Austrians, several types of selection biases are excluded.

Taken together, this real-world analysis showed a considerably higher sex-specific mortality in men in the first 30 days after bariatric surgery as well as a higher overall mortality rate in men compared to women. Moreover, in patients deceased after bariatric surgery, cardiovascular and psychiatric comorbidities were frequently observed, with no relevant sex-specific differences. However, diabetes rates were higher in men and malignancies were predominant in women. In conclusion, the higher age at operation in men together with a higher intake of cardiovascular drugs and antidiabetics preoperative highlight the importance of an individualized, sex-specific preoperative counseling before bariatric surgery. Further studies are warranted to evaluate if earlier timing of bariatric surgery in men might improve mortality outcomes.
